# Association of brain white matter microstructure with cognitive performance in major depressive disorder and healthy controls: a diffusion-tensor imaging study

**DOI:** 10.1038/s41380-021-01330-8

**Published:** 2021-10-25

**Authors:** Susanne Meinert, Nico Nowack, Dominik Grotegerd, Jonathan Repple, Nils R. Winter, Isabel Abheiden, Verena Enneking, Hannah Lemke, Lena Waltemate, Frederike Stein, Katharina Brosch, Simon Schmitt, Tina Meller, Julia-Katharina Pfarr, Kai Ringwald, Olaf Steinsträter, Marius Gruber, Igor Nenadić, Axel Krug, Elisabeth J. Leehr, Tim Hahn, Katharina Thiel, Katharina Dohm, Alexandra Winter, Nils Opel, Ricarda I. Schubotz, Tilo Kircher, Udo Dannlowski

**Affiliations:** 1 Institute for Translational Psychiatry, University of Münster, Münster, Germany; 2 Institute for Translational Neuroscience, University of Münster, Münster, Germany; 3 Department of Psychiatry and Psychotherapy, University of Marburg, Marburg, Germany; 4 Center for Mind, Brain and Behavior (CMBB), Philipps-Universität Marburg and Justus Liebig Universität Giessen, Hans-Meerwein-Str. 6, 35032 Marburg, Germany; 5 Department of Psychiatry, Social Psychiatry, and Psychotherapy, Hannover Medical School, Hannover, Germany; 6 Department of Psychiatry and Psychotherapy, University Hospital Bonn, Bonn, Germany; 7 Department of Psychology, University of Münster, Münster, Germany

**Keywords:** Diagnostic markers, Neuroscience

## Abstract

Cognitive deficits are central attendant symptoms of major depressive disorder (MDD) with a crucial impact in patients’ everyday life. Thus, it is of particular clinical importance to understand their pathophysiology. The aim of this study was to investigate a possible relationship between brain structure and cognitive performance in MDD patients in a well-characterized sample. *N* = 1007 participants (*N*_MDD_ = 482, healthy controls (HC): *N*_HC_ = 525) were selected from the FOR2107 cohort for this diffusion-tensor imaging study employing tract-based spatial statistics. We conducted a principal component analysis (PCA) to reduce neuropsychological test results, and to discover underlying factors of cognitive performance in MDD patients. We tested the association between fractional anisotropy (FA) and diagnosis (MDD vs. HC) and cognitive performance factors. The PCA yielded a single general cognitive performance factor that differed significantly between MDD patients and HC (*P* < 0.001). We found a significant main effect of the general cognitive performance factor in FA (*P*_*tfce-FWE*_ = 0.002) in a large bilateral cluster consisting of widespread frontotemporal-association fibers. In MDD patients this effect was independent of medication intake, the presence of comorbid diagnoses, the number of previous hospitalizations, and depressive symptomatology. This study provides robust evidence that white matter disturbances and cognitive performance seem to be associated. This association was independent of diagnosis, though MDD patients show more pronounced deficits and lower FA values in the global white matter fiber structure. This suggests a more general, rather than the depression-specific neurological basis for cognitive deficits.

## Introduction

Cognitive deficits are attendant symptoms of major depressive disorder (MDD) as defined by the International Classification of Disease (ICD-11) [1] that occur in two-thirds of depressed patients [2]. Deficits were described in several domains of cognition including executive function, attention, concentration, learning, memory, and psychomotor processing speed [2–5], while automatic stages of processing seem to be less affected than controlled, effortful processing domains [6, 7].

Understanding the causes of cognitive dysfunction in depression is of high clinical relevance. Some cognitive deficits seem to persist after remission [8–10], and increase with every MDD episode [11, 12]. They are associated with reductions in psychosocial functioning in MDD [13, 14] with consequences for occupation, social interactions, and health. First, patients suffering from cognitive deficits are less likely to obtain and sustain a job [15]. Second, they have problems maintaining household, or social and family relationships [16]. Lastly, cognitive deficits appear to increase proneness to relapse [17] and suicidal ideations [18], by reducing social support [16], impairing treatment success [19], and compromising problem-solving capacities [20]. The treatment of cognitive deficits could therefore improve MDD patients’ functioning.

The neurobiological perspective might shed light on the underpinnings of cognitive deficits in MDD. Following lesion studies, the classic neurobiological view of cognitive deficits focused on the impairment of specialized brain regions responsible for unique cognitive operations. In doing so, complex models were established that focus on the prefrontal cortex, hippocampus, anterior cingulate cortex, and basal ganglia [21].

The brain is, however, characterized by a network of complex, reciprocal anatomical connections. Thus, the connectome perspective that higher cognitive functioning depends upon the integration of various inputs from specialized regions, seems more consistent with the brain’s architecture [22]. One possible measure of the microstructure of interconnecting fibers is diffusion-tensor imaging (DTI), a noninvasive, affordable, and efficient measurement to estimate fiber microstructure, reflecting myelination, axon density, axon diameter, and the number of fibers [23, 24]. White matter microstructure assessed by means of DTI has shown strong associations with cognitive performance in multiple studies in healthy controls (HC) and patient groups. In HC, cognitive performance measures were linked to fiber integrity in frontal association fibers [25–27], like the corpus callosum (CC), the cingulum bundle (CB), the superior longitudinal fasciculi (SLF), or the inferior fronto-occipital fasciculi (IFOF) [28, 29]. Likewise, similar associations of DTI-based measures of fiber integrity and cognitive deficits have already been described in different brain disorders, e.g., stroke [30], Parkinsons disease [31], small-vessel disease [32], multiple sclerosis [33], diabetes [34], substance abuse [22], schizophrenia [35, 36], or bipolar disorder [37].

However, the role of white matter integrity regarding cognitive deficits of MDD patients has attracted less attention, albeit MDD was associated with reductions in fiber microstructure in the IFOF, the uncinate fasciculi (UF), the thalamic radiation (TR), the corticospinal tract (CT), and the inferior longitudinal fasciculi (ILF) and the SLF, the CB, and the CC compared with HC [38–41] and changes in the white matter connectome [42, 43].

Unfortunately, studies investigating the association between these microstructural abnormalities and cognitive deficits in MDD are sparse: In geriatric depression, associations between cognitive deficits and brain microfiber structure were found in overall prefrontal white matter, the CC, the TR, and the UF [44–46]. However, we are not aware of a study investigating white matter disturbances using DTI over the entire age and severity range of MDD patients.

Another open question is the specificity of these potential alterations in MDD. The aim of this study was, thus, to the extent of previous results to the entire severity spectrum of MDD patients and to compare the association between fiber microstructure and cognitive deficits with HC. First, we expect MDD patients to perform worse on cognitive tests. These deficits should decline, but still be detectable in remitted patients (hypothesis 1). Second, we expect that MDD patients have lower fiber microstructure compared with HC in the IFOF, the UF, the TR, the CT, the SLF and the ILF, the CB, and the CC (hypothesis 2). Further, as associations between white matter microstructure and cognitive deficits were already shown for a wide range of disorders, we do not expect that the association of cognitive test measures and white matter integrity is restricted to MDD patients. Rather, we would assume that the magnitude of the association between white matter and cognitive functioning should be similar between MDD patients and HC (hypothesis 3).

## Materials and methods

### Participants

*N* = 1007 participants (MDD: *N* = 482, *M*_age_ = 37.12, 311♀, HC: *N* = 525, *M*_age_ = 31.68, 321♀, Table 1, Supplement 1) were selected from the FOR2107 cohort assessed at two scanning sites—Marburg and Münster (the general description of the study [47] and the magnetic resonance imaging (MRI) quality-assurance protocol [48] are provided elsewhere). Participants were recruited through newspaper advertisements or in psychiatric hospitals.

**Table 1:** Descriptive statistics of the sample used in this study.

	MDD (*N* = 482)	HC (*N* = 525)	Test statistic	*P* value	Cohen’s *d*
Age, M ± SD	37.12 ± 13.47	31.68 ± 11.87	*t*(962.3) = −6.77^a^	<0.001	0.436
Sex, f/m	311/171	321/204	χ²(1) = 1.23^b^	0.268	–
IQ_MVT_, M ± SD	113.79 ± 13.71	114.98 ± 13.72	*t*(1005) = 1.38^c^	0.170	0.087
Education years, M ± SD	13.15 ± 2.76	13.98 ± 2.42	*t*(1005) = 5.05^c^	<0.001	0.329
BDI Sum, M ± SD	17.99 ± 11.19	2.50 ± 2.15	*t*(505.68) = −29.68^a^	<0.001	2.640
TMT-A, M ± SD	26.16 ± 10.36	22.52 ± 8.40	*t*(927.0) = −6.10^a^	<0.001	0.401
TMT-B, M ± SD	57.12 ± 24.30	46.89 ± 17.30	*t*(861.5) = −7.64^a^	<0.001	0.521
DSST, M ± SD	55.97 ± 12.17	65.45 ± 11.05	*t*(973.2) = 12.9^a^	<0.001	0.827
RAVLT-S, M ± SD	55.73 ± 9.87	60.15 ± 8.27	*t*(941.7) = 7.68^a^	<0.001	0.501
RAVLT-R, M ± SD	13.14 ± 2.99	13.90 ± 1.89	*t*(799.2) = 4.75^a^	<0.001	0.336
CBTT-f, M ± SD	8.65 ± 1.84	9.44 ± 1.96	*t*(1005) = 6.63^c^	<0.001	0.418
CBTT-b, M ± SD	8.04 ± 1.91	9.00 ± 1.71	*t*(969.0) = 8.42^a^	<0.001	0.541
d2, M ± SD	168.05 ± 43.05	194.00 ± 42.86	*t*(1005) = 9.58^c^	<0.001	0.604
LNS, M ± SD	15.83 ± 3.20	16.96 ± 3.09	*t*(1005) = 5.70^c^	<0.001	0.360
VF-C, M ± SD	23.20 ± 6.01	25.45 ± 5.63	*t*(1005) = 6.15^c^	<0.001	0.388
VF-P, M ± SD	11.32 ± 4.22	12.44 ± 4.41	*t*(1005) = 4.11^c^	<0.001	0.259
VF-A, M ± SD	15.27 ± 3.48	16.90 ± 3.26	*t*(1005) = 7.62^c^	<0.001	0.481
General Cognitive Performance factor, M ± SD	−0.37 ± 1.03	0.34 ± 0.84	*t*(1005) = −12.04	<0.001	0.935
Number of hospitalizations, M ± SD	1.66 ± 2.16	–	–	–	–
Medication Load Index, M ± SD	1.32 ± 1.43	–	–	–	–
Comorbid diagnosis (yes/no)	203/279	–	–	–	–

*BDI Sum* beck depression inventory, *CBTT-f/b* Corsi block-tapping test, forwards/backwards, *d2* d2 test of attention, *DSST* digit symbol substitution test, *HC* healthy control, *IQMVT* Intelligence quotient evaluated with the multiple-choice vocabulary test version B (dt. “Mehrfachwahl-Wortschatz-Test Version B”), *LNS* letter–number–sequences test, *M* mean, *MDD* major depressive disorder, *RAVLT-S/B* Rey Auditory Verbal Learning Test, sum of all correct words/recognition, *SD* standard deviation, *TMT-A/B* trail making test, Version A/B, *VF-C/P/A* verbal fluency test, category/phonemic/alternating.

^a^Two-sample *t* test assuming unequal variance, ^b^Pearson χ² test, ^c^two-sample *t* test assuming equal variance.

The FOR2107 cohort was approved by the Ethics Committees of the Medical Faculties, University of Marburg and University of Münster. All experiments were performed in accordance with the ethical guidelines and regulations. All participants gave written informed consent prior to examination. They received financial compensation for participation after the testing session.

Trained personnel confirmed psychiatric diagnoses or the lack thereof using the Structural Clinical Interview for DSM-IV-TR (SCID-IV) [49]. MDD patients were considered if they reported a current or lifetime diagnosis of MDD (severe, moderate, mild, (partially) remitted episode). Remission was defined as the absence of DSM-IV-TR diagnostic criteria for a MDD episode for at least two months at the time of the interview. Partial remission classifies patients with subclinical symptoms (i.e., symptoms are insufficient to fulfill the diagnostic criteria of an MDD episode but severe enough to interfere with daily functioning) or if the time of recovery was shorter than 2 months.

### Questionnaires, tests, and other clinical characteristics

In the FOR2107 cohort, all participants underwent neurocognitive testing in five subdomains of cognition: (1) executive functioning and sustained attention, 2) long and short-term memory performance, (3) visuospatial working memory, (4) verbal working memory, and (5) semantic processing. For a detailed description of the neurocognitive test battery, see Supplement 2. The general intelligence quotient (IQ_MVT_) was estimated with the German version of the multiple-choice vocabulary intelligence test (MVT). Participants provided their highest educational degree. Education years were then estimated according to the typical time it takes to acquire the said degree.

To correct for typical clinical characteristics associated with MDD the following questionnaires and scores were used: The Beck Depression Inventory (BDI) [50] to assess current symptomatology, the number of prior hospitalizations provided by the participants in an interview, the Medication Load Index [51], a composite measure of total medication load reflecting daily dose and number of prescriptions irrespective of active components, and the presence of any comorbidities provided by the SCID-I interview.

### Analysis

#### Analysis 1: factor analysis

To address hypothesis 1, we conducted an explorative principal component analysis (PCA; KMO = 0.888; Bartlett (66) = 4308.3, *P* < 0.001) with varimax rotation to reduce the 12 neuropsychological test scores to fewer variables. The component scores for each extracted factor were computed using a regression approach. Demographic data and the component analysis were analyzed using IBM SPSS Statistics 26 (SPSS Inc., Chicago, IL, USA).

#### Analyses 2 and 3: diffusion-tensor imaging

The DTI data acquisition, quality-assurance protocol, and preprocessing steps have already been published [38]. Detailed information can be found in Supplements 3. Analysis was performed with FSL5.0.10 (http://fsl.fmrib.ox.ac.uk/fsl/fslwiki/, FMRIB, Oxford Center for Functional MRI of the Brain, University of Oxford, Department of Clinical Neurology, John Radcliffe Hospital, Oxford, UK) [52–54]. Tensor-derived maps were generated and fractional anisotropy (FA), mean diffusivity (MD), radial diffusivity (RD), and axial diffusivity (AD) for each voxel per participant were estimated [55]. FA is defined as the normalized variance of the three eigenvalues about their mean. FA quantifies directional diffusion, MD is the average of all three eigenvalues, AD is equivalent to the first eigenvalue reflecting the primary diffusion direction which representing tract orientation, and RD is the mean of the second and third eigenvalue, representing motion perpendicular to the tract. As the number of fibers, fiber crossings, and general fiber orientation can also influence diffusion metrics in healthy fiber structure [56], the values should be interpreted with caution. Nonetheless, increased MD and decreased FA are measures of neuronal injury, while increased RD measures demyelination and increased AD axonal damage [57].

As tract-based spatial statistics (TBSS) reduces partial volume effects and registration misalignments [58], it was used for all DTI analyses (Supplements 3). To test for statistical significance, the nonparametric permutation testing implemented in FSL’s “randomize” [59] was used with 5000 permutations. Using the default options optimized for TBSS, threshold-free cluster enhancement (TFCE) was used to correct for multiple comparisons. Significance was determined by correcting for the family-wise error (FWE; *P* < 0.05) using the 95th percentile of the null distribution of permutated input data of the maximum TFCE scores [60]. For scatterplots and additional analyses in SPSS, the average FA per participant of significant clusters was extracted using FSL’s “fslstats”. The total intracranial volume (TIV) was extracted from T_1_ images using the Computational Anatomy Toolbox (CAT-12, http://www.neuro.uni-jena.de/cat, v933, Supplement 3). More detailed information about the statistics and general methods can be found in Supplement 4.

Results focus on FA as it is the most widely reported DTI measure. However, as the combination of different DTI metrics can be beneficial for interpretation, results in MD, RD, and AD are described in Supplement 5. To correct for scanner differences, two dummy coded variables (Marburg pre-body-coil change, Marburg post-body-coil change) with Münster as reference category were calculated.

To replicate the effects of reduced fiber integrity (lower FA, higher MD/RD) in MDD patients compared with HC (hypothesis 2) an ANCOVA with FA as the dependent variable, diagnosis (HC vs. MDD) as an independent variable was conducted (Analysis 2).

Further, to investigate the association of neurocognitive functioning with fiber structure, an ANCOVA with FA as the dependent variable, diagnosis (HC vs. MDD), the extracted neurocognitive factors, and their interaction with diagnosis as independent variables was conducted (Analysis 3). We expected a significant main effect of neurocognitive functioning irrespective of diagnosis (Hypothesis 3). Additional control analyses were performed including a complementary tractography-based connectome analysis and analyses using T_1_ structural data (Supplement 3). If not otherwise specified, all analyses included the following nuisance variables: age, sex, TIV, Marburg pre-body-coil, Marburg post-body-coil, IQ_MVT_, and the number of education years.

## Results

### Analysis 1. Factor analysis for neuropsychological tests

As expected, MDD patients performed significantly worse compared to HC on all neuropsychological tests (all *P* < 0.001, Table 1) with small (e.g., for verbal fluency) to large (processing speed) effect sizes (Cohen’s *d* range:[0.26–0.83]). While the five-factor structure ((1) executive functioning and sustained attention, (2) long and short-term memory performance, (3) visuospatial working memory, (4) verbal working memory, and (5) verbal fluency) could be replicated with clear allocations of each test to one of the five factors (Supplement 6), the scree plot (Supplementary Fig. 1) from the explorative PCA strongly suggested a one-factor solution (EV_factor_ = 4.953) accounting for 41.28% of the variance and factor-loadings ranging from 0.461 to 0.777 (Supplementary Table 1).

#### Differences between MDD and HC in cognitive performance

**Fig. 1:**
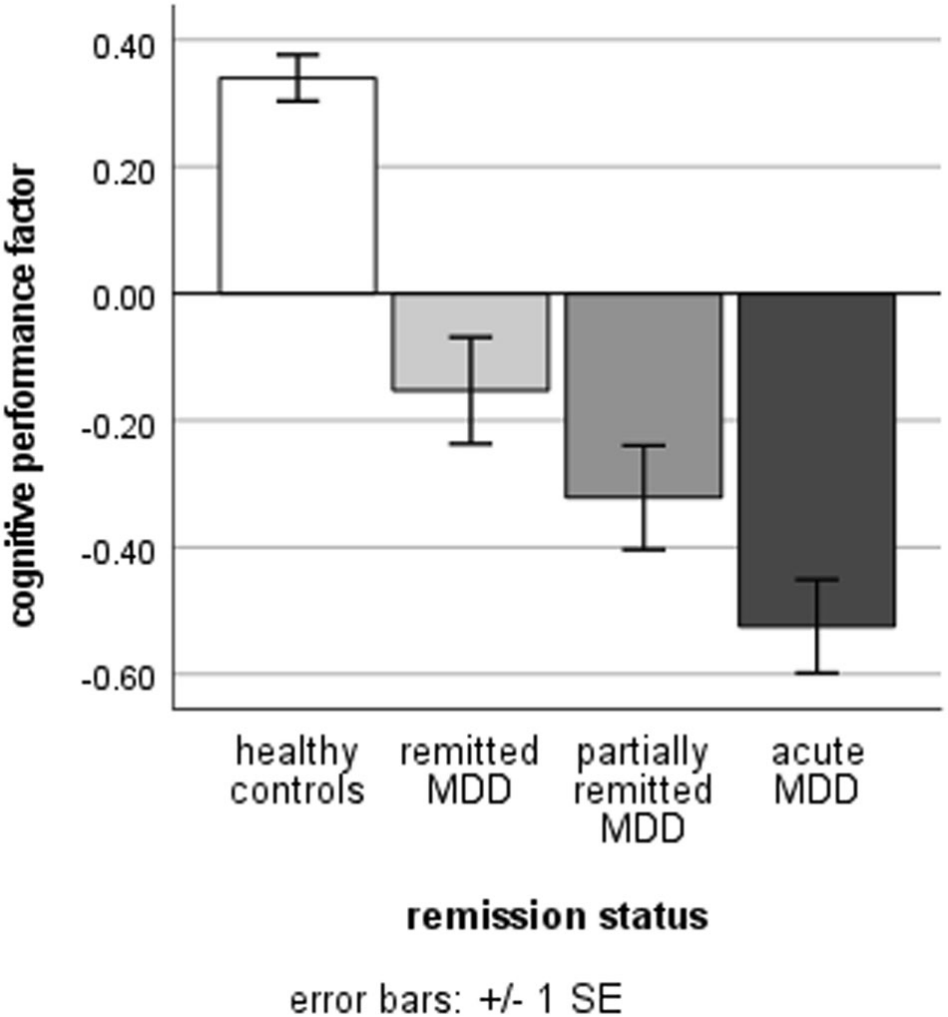
The general cognitive performance factor in HC and MDD patients. MDD patients were divided into remitted, partially remitted and acute MDD by the SCID-I diagnoses. HC   healthy controls, MDD   major depressive disorder, SE   standard error.

GCP differed significantly between MDD patients and HC (*F*(1,1001) = 74.46, *P* < .001, *η*² = 0.069) with a medium sized effect even after taking age, sex, IQ_MVT_ and education years into account. More precisely, acute and (partially) remitted MDD patients differed from HC in their GCP (*F*(1,1001) = 34.40, *P* < 0.001, *η*² = 0.033, Fig. 1). Post hoc Bonferroni corrected tests revealed that HC differed from acute (*P* < 0.001, 95% confidence interval (CI): [0.67, 1.06]), partially remitted (*P* < 0.001, CI: [0.42, 0.91]) and completely remitted MDD patients (*P* < 0.001, CI: [0.25, 0.73]), respectively. Acute MDD patients presented with lower GCP compared with completely remitted MDD patients (*P* = 0.002, CI: [−0.64, −0.10]), but not compared with partially remitted MDD patients (*P* = 0.300, CI: [−0.48, 0.07]. Lastly, the difference between partially and completely remitted MDD patients was not significant (*P* = 0.877, CI: [−0.48, 0.14]). Likewise, GCP was negatively associated with depression severity (BDI) in the MDD subsample after controlling for age, sex, IQ_MVT_, and education years (*F*(1,469) = 4.82, *P* = 0.029, *η*² = 0.010; *b* = −0.007).

### Analysis 2. Group differences (MDD vs. HC)

**Fig. 2:**
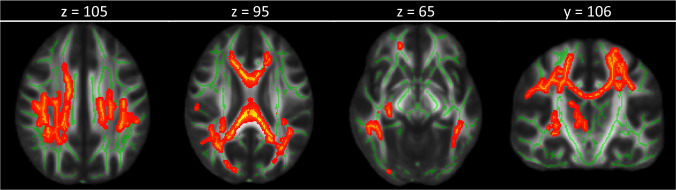
Healthy controls had higher fractional anisotropy (FA) values compared with acute depressive patients. FA values are displayed at *P*_*tfce-FWE*_ < 0.05 onto the FMRIB58 template. Slice position is noted above the brain images.

Prior to the inclusion of GCP in the model, the effect of diagnosis on FA was not significant (*P*_*FWE*_ = 0.072). Significant effects were found for AD, but not in MD and RD (Supplement 2). However, when including only acute MDD patients and HC, a main effect of diagnosis was found in FA (*P*_*tfce-FWE*_ = 0.018, k = 15,111 voxels in three clusters, MNI-coordinates of the peak voxel from the largest cluster: *x* = 34, *x* = −18, 36, Fig. 2) in all eight anticipated fiber bundles (Supplementary Table 2).

### Analysis 3. ANCOVA including the general cognitive performance (GCP) factor

**Fig. 3:**
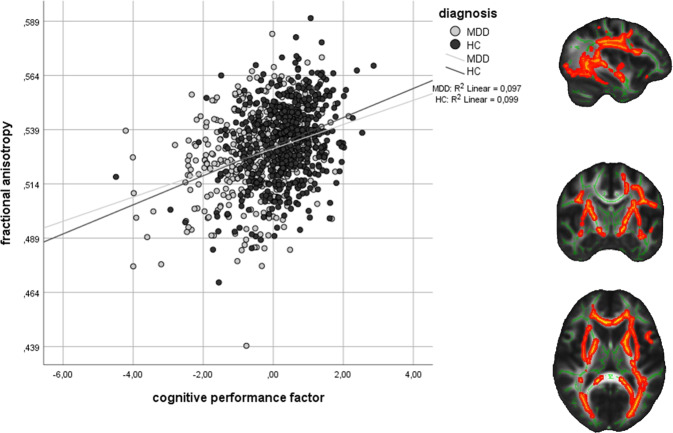
Association of fractional anisotropy (FA) and the general cognitive performance factor in major depressive disorder (MDD) patients and healthy controls (HC). FA values were extracted with a threshold of *P*_*tfce-FWE*_ < 0.01 and displayed onto the FMRIB58 template in the *x* = −36, *y* = −9, *z* = 11 planes in MNI space. The scatterplot depicts mean FA values of the significant cluster with a threshold of *P*_*tfce-FWE*_ < 0.05.

After including GCP into the model, neither the main effect of diagnosis (*P*_*tfce-FWE*_ = 0.264) nor a diagnosis × GCP interaction (*P*_*tfce-FWE*_ = 0.365) could be found for FA, while a significant main effect of diagnosis was still present for AD (Supplement 5). However, we found a significant main effect of GCP (*P*_*tfce-FWE*_ = 0.002, *k* = 43,700 voxels in one cluster, MNI-coordinates of the peak voxel: *x* = 31, *y* = −66, *z* = 11, Supplementary Table 3, Fig. 3) in a large bilateral cluster consisting of the CC, the IFOF, the anterior TR, and the SLF and ILF among other regions (Supplementary Table 2). Even after excluding (partially) remitted MDD patients to reduce variance and enhance differences with HC, this association between FA and GCP remained (Supplement 7). To verify that results were independent of the MRI scanner, two ANCOVAs with the mean FA values per participant from the significant cluster as the dependent variable were calculated in SPSS. This analysis confirmed that the GCP effect was present at both scanning sites, respectively (Marburg: *F*(1,628) = 31.67, *P* < 0.001, *η*² = .048, Münster: *F*(1,361) = 12.93, *P* < 0.001, *η*² = 0.035). As GCP and diagnosis are depending on each other, a linear ANCOVA might not be adequate to disentangle their effects. Thus, the analysis was repeated in SPSS using a nonparametric Generalized estimating equation (GEE) analysis. The results were confirmed using this method (main effect diagnosis: Wald-χ²(1) = 0.29, *P* = 0.589; main effect GCP: Wald-χ²(1) = 25.95, *P* < 0.001; interaction: Wald-χ²(1) = 0.40, *P* = 0.528). The tractography-based connectome analysis confirmed that the associations with GCP were widespread, including fibers connecting nearly all anatomical brain regions irrespective of the method of analysis (Supplement 8 and Supplementary Fig. 2). The effect in the MDD subgroup remained significant, even after taking medication intake, the presence of comorbid diagnoses, number of previous hospitalizations, and BDI into account in an additional ANCOVA in SPSS (Table 2). Except for verbal working memory performance, all subdomains of GCP were associated with the mean extracted FA from the significant cluster. The strongest effects were found for processing speed and sustained attention (Supplementary Table 4). A significant association with GCP was also present for MD and RD (Supplement 3). There was no positive correlation between GCP and gray matter volume at *P*_*tfce-FWE*_ = 0.217.

**Table 2:** Using only the participants with MDD diagnosis, an ANCOVA was calculated with mean extracted FA values as the independent variable and medication intake, presence of a comorbid diagnosis, number of hospitalizations, and acute symptomatology on top of age, sex, TIV, scanner/side variables, IQ_MVT_ and number of education years in SPSS.

Factor/covariate	*F*-statistic, df(1,459)	*P* value	*η*²
General cognitive performance	12.611	<0.001	0.027
Age	24.773	<0.001	0.051
Sex	1.476	0.225	0.003
TIV	38.753	<0.001	0.078
Marburg pre-body-coil	71.228	<0.001	0.134
Marburg post-body-coil	43.299	<0.001	0.086
IQ_MVT_	1.081	0.299	0.002
Number of education years	3.821	0.051	0.008
Medication Load Index	0.835	0.361	0.002
Comorbid disorder	0.275	0.600	0.001
Number of hospitalizations	0.486	0.486	0.001
BDI	0.280	0.597	0.001

*ANCOVA*   analysis of covariance, *BDI*   Beck’s Depression Inventory Score, *FA*   fractional anisotropy, *IQ*_*MVT*_   intelligence quotient evaluated with the multiple-choice vocabulary test version B (dt. “Mehrfachwahl-Wortschatz-Test Version B”), *MDD*   major depressive disorder, *TIV*   total intracranial volume.

## Discussion

The aim of this study was to investigate the association between white matter fiber microstructure and cognitive deficits in MDD patients over the entire spectrum of the disorder. As expected, general cognitive performance was associated with FA in a large bilateral cluster consisting of the CC, the IFOF, the anterior TR, and the SLF and ILF among other regions. This effect seems to be driven by deficits in processing speed and sustained attention, semantic processing, and memory, while small (visuospatial working memory) or no associations (verbal working memory) could be found for working memory. The associations between fiber microstructure and general cognitive performance were very robust even after correction for general intelligence, educational achievement, medication intake, and presence of comorbid diagnoses, number of previous hospitalizations, and BDI in the MDD subsample. They were confirmed using a different method of analysis (tractography-based connectome analysis), and were found at both MRI scanners, respectively. Lastly, similar associations were found for other DTI measures of fiber integrity (MD and RD). While the interpretation of single DTI measures is limited [56], the combination of reduced FA and increased MD and RD values might suggest neuropathological processes as the basis for cognitive deficits. This effect seems to be confined to white matter microstructure and not gray matter volume, as no positive associations were found between cognitive performance and gray matter structural MRI data.

These results highlight that intact fiber microstructure is associated with fast and accurate communication between brain regions required for optimal cognitive functioning [22]. Previous studies have already postulated that fine-tuned prefrontal signaling—with too much or too little signaling reducing cognitive performance—could be fundamental for sustained attention [61]. If the communicating fibers between frontal areas and other brain regions are structurally impaired, as hinted at by reduced FA in those fibers, this could result in cognitive impairment. It must be noted, however, that the results in this study seem to be regionally unspecific, as multiple tracts in a large bilateral cluster were affected.

Second, we found that MDD patients performed consistently worse on all cognitive tasks in concordance with previous analyses [2]. While these effects were found most strongly in tests assessing processing speed, a wide range of cognitive processes were affected in MDD patients, reflected by the single general cognitive performance factor extracted in the PCA. The differences between HC and acute or (partially) remitted MDD patients, respectively, support and extent the well-known report that cognitive deficits in MDD—while being alleviated—seem to persist in remission [14]. Deficits in cognitive performance could influence the inhibition of inappropriate behavioral or emotional processes, planning of future behavior, and flexible problem solving [14]. Cognitive deficits in remitted MDD patients may play a crucial role in sustaining psychosocial functioning—socially, mentally as well as in more general societal functions like workplace productivity. The neurobiological underpinnings of FA reductions associated with cognitive performance in MDD patients are most likely complex. It is possible, that interactive effects of genetic influences and environmental stressors might result in more pronounced fiber integrity reductions and, hence, elevated cognitive deficits in MDD [8, 62]. The variation in time of white matter maturation differs regionally. Especially association and commissural white matter fibers responsible for higher cognitive functioning, continue to develop throughout adolescence to early and middle adulthood [36, 63]. This prolonged development is the underlying basis for white matter plasticity, which in turn would be necessary for environmental and (epi-)genetic factors to in turn influence fiber structure [63–65].

Third, in contrast to our hypothesis, MDD patients’ FA differed only marginally from HC. Previous studies [38, 66] have already drawn into question that MDD patients’ microstructure differs from HC on a general basis. Choi et al. argued that small sample sizes, tracts prone to artifacts, or other aspects of MDD pathology (e.g., course of illness, childhood maltreatment experiences, antidepressant treatment, or specific symptoms) could have produced the significant differences between MDD patients and HC in earlier studies [66]. Likewise, the significant reduction in FA values in acute MDD patients (i.e., those who take more medication and experience more severe symptoms) compared with HC in our well-powered analysis suggests that the lifetime MDD diagnosis by itself might not be the sole driving force of white matter alterations in patients.

Acute MDD patients take more psychiatric medications and have a worse course of illness than (partially) remitted MDD patients in our sample, this could explain this difference.

Future studies might use the results of this study to use white matter morphological abnormalities associated with cognitive deficits in MDD to guide the inquiry of new therapeutic options. It should be investigated whether current treatment options for cognitive deficits in MDD [3, 8, 67, 68] like biobehavioral interventions (e.g., exercise, sleep hygiene, healthy diet), pharmacological treatments (e.g., vortioxetine), neurostimulation techniques (e.g., transcranial magnetic stimulation), and psychotherapeutic interventions (e.g., cognitive behavioral therapy, cognitive remediation therapy) can be linked to changes in MDD’s fiber structure.

### Limitations

Some limitations should be acknowledged. First, while DTI is a feasible and noninvasive technique, FA can also be influenced by other, non-pathological factors, e.g., the number and orientation of axons irrespective of fiber damage [56]. Second, while we tried to account for influences attributed to current psychiatric medication intake, the influence of therapy (including psychotherapy, electroconvulsive therapy, etc.), cannot be ruled out completely. Therefore, future studies should try to replicate the findings of this study in untreated MDD patients or systematically investigate the influences of treatment. Third, while MDD patients and HC did not differ in their IQ_MVT_ scores, we found a significant difference in their educational achievements. We have to note that, while the MVT is a robust and efficient estimator for general IQ, it is also prone to slight measuring inaccuracies [69] and captures crystallized intelligence rather than fluid intelligence. Hence, even after correcting for educational years and IQ_MVT_ confounding effects of general intelligence cannot be ruled out entirely. Lastly, this study is correlational, and cannot evaluate causal effects. To this end, longitudinal studies are needed that investigate fiber microstructural changes over the course of MDD or pre-post treatment. Regardless, the major strength of this study is the use of a large, well-characterized sample reflecting the entire spectrum of MDD patients.

## Conclusion

Our findings highlight the importance of neurobiological wiring in cognitive performance in healthy controls and MDD patients. They provide robust evidence that global structural connectivity is associated with cognitive performance in MDD patients and HC. This association was independent of diagnosis, suggesting a general association between DTI measures of fiber integrity and cognitive performance. Efforts to treat cognitive deficits in MDD should, thus, consider the white matter as one of the underlying neural mechanisms.

## Supplementary information

Supplementary Material
