# Supplementary material for: Association of brain white matter microstructure with cognitive performance in major depressive disorder and healthy controls: a diffusion-tensor imaging study
Source: Mol Psychiatry. 2021 Oct 25;27(2):1103–10. doi: 10.1038/s41380-021-01330-8 (PMC9054669; doi:10.1038/s41380-021-01330-8)
Supplement: Supplementary file 1 — Supplementary Material [file 41380_2021_1330_MOESM1_ESM.docx]

**Supplements**

**Supplement 1: Choosing participants from the FOR2107 cohort**

The second data freeze (26.02.2019) was used with N=733 HC and N=629 MDD. Caucasian participants varying in age from 18-65 years were recruited. Pre-established exclusion criteria comprised any neurological abnormalities, history of seizures, head trauma or unconsciousness, severe physical impairment, severe craniocerebal injury, hypothyroidism without adequate medication, claustrophobia, color blindness, and general MRI contradictions. Lifetime diagnoses of schizophrenia, schizoaffective disorder, bipolar disorder, or substance dependence were excluded. More precisely, participants with DTI (N=716 HC, N=601 MDD), without a diagnosis of substance dependence, an age between 18 and 65 years, without severe physical impairment or craniocerebral injury were included, on top of participants who were compliant (N=694 HC, N=573 MDD), with Caucasian origin (N=644 HC, N=518 MDD), and who survived the T1 and DTI quality insurance and had no pathological neuronal changes (N=635 HC, N=506 MDD). HC had to have a BDI score smaller than 8 and no prior hospitalizations (N=535 HC, N=506 MDD), as the SCID-I diagnosis did not seem reliable otherwise. Participants had to complete all the necessary questionnaires and interviews, resulting in the final sample of N=525 HC and N=482 MDD.

**Supplement 2: The neurocognitive test battery**

To assess executive functioning and sustained attention over continuous performance tasks three tests were used: 1. the Digit Symbol Substitution Test (DSST)^1^, a subtest of the Wechsler Adult Intelligence Scale – Revised^2,3^ in the 90sec version. This subtest uses a series of numbers, each of them paired with an abstract symbol. Subjects must draw the symbols under the corresponding numbers using a coding key. The test score is calculated counting the number of correctly completed pairs. 2. The Trail-Making-Test (TMT)^4,5^, in which participants are instructed to connect a set of 25 dots as quickly as possible while maintaining accuracy. It consists of two parts. In the first part (TMT-A) targets are all numbers that need to be connected in sequential order. In the second part (TMT-B) the subjects need to alternate between numbers and letters both in sequential order. 3. The d2 Test of Attention (d2)^6^ was used to assess selective and sustained attention. Participants are required to cross out the letter “d” with two marks below or above it in a list of distractors (letter “p”, one, three or four marks).

To assess memory performance the Rey Auditory Verbal Learning Test (RAVLT)^7,8^ was employed. In this test, participants are given a list of 15 unrelated words and are asked to repeat. This is repeated over five trials. After 30 minutes the participants are asked again to recognize them in a list of distractors. We measured overall memory performance (sum of all correct words over the 5 trials; RAVLT-S) as an approximation of short-term memory and recognition (RAVLT-R) performance for long-term memory performance.

The Corsi block-tapping test (CBTT)^9,10^ was used to assess visuospatial working memory performance. Participants are instructed to mimic the tapping sequence of up to nine identical spatially separated blocks by the instructor. In the first trial participants are instructed to follow the same direction as the instructor (CBTT-f), while in the second trial participants need to tap in backward order (CBTT-b).

The German version of the Letter-Number-Sequences (LNS), again, a subtest of the Wechsler Adult Intelligence Scale^2^ was used to test verbal working memory performance. Participants need to recall numbers in increasing and letters in alphabetical order after they have been red to them in mixed sequences. We counted the number of correctly recalled sequences.

Lastly, to assess semantic processing the verbal fluency (VF) test^11^ was used. Participants were asked to name as many animals (VF-C), words beginning with the letter “p” (VF-P) or fruits and sports in alternating order (VF-A) as possible.

The general intelligence quotient (IQ_MVT_) was estimated with the German version of the multiple-choice vocabulary intelligence test (dt. “Mehrfachwahl-Wortschatz-Intelligenztest”, MVT)^12,13^. Participants are presented with a list of 37 target words and four distractors each. Participants have to choose the one target word which is an authentic, German word from the dictionary. The amount of correct words is used to estimate intelligence.

**Supplement 3: DTI and T_1_ data acquisition, and image processing**

Data was acquired using a 3T whole body MRI scanner. In Marburg a Siemens Magnetom Trio Tim syngo MR B17, Erlangen, Germany with a 12-channel head matrix Rx-coil was used. In Münster data was collected using a Siemens 3T PRISMA, Erlangen, Germany, with a 20-channel head matrix Rx-coil.

**DTI data acquisition.** For both scanners, a GRAPPA acceleration factor of 2 was used for 56 axial slices, 2.5mm thick with no gap, with an isotropic voxel size of 2.5x2.5x2.5mm³ (TE=90ms, TR=7 300ms). Five non-diffusion weighted (DW) images (*b_0_*=0) and 2x30 DW images with a *b*-value of 1000sec/mm² were acquired. To assure data quality the open-source software DTIPrep^14^ was used. The included participants had 64.30 images on average (*SD*=1.23, range: 54-65).

**DTI preprocessing.** Preprocessing was performed with FSL5.0.10 (http://fsl.fmrib.ox.ac.uk/fsl/fslwiki/, FMRIB, Oxford Center for Functional MRI of the Brain, University of Oxford, Department of Clinical Neurology, John Radcliffe Hospital, Oxford, United Kingdom)^15–17^. The DW images were corrected for eddy and motion artifacts using FSL’s “eddy”^18^ and *b*-vectors were rotated after eddy correction. Following automated skull stripping using FSL’s brain extraction tool (BET)^19^, the first *b_0_* was used as reference for alignment. For diffusion tensor estimation “DTIFIT” within FMRIB’s Diffusion Toolbox (FDT) was used.

**TBSS preprocessing and analysis.** All images were registered to the FMRIB58_FA template (1x1x1mm³ Montreal Neurological Institute (MNI) standard space). All images were resliced to 1x1x1mm³ image space. A white matter skeleton was created for the aligned fractional anisotropy (FA) images (threshold 0.2) to mask each participant’s registered FA image. For group-level comparisons, the maximum weighted for distance FA value orthogonal to the skeleton was moved to skeleton space. Cluster sized and peak voxels MNI coordinates were retrieved using FSL’s “cluster” tool. Anatomical labels of significant clusters corresponding to the “JHU White-Matter Tractography Atlas”^20–22^ were extracted using FSL’s “atlasquery”.

**Connectome reconstruction.** As TBSS analyses lack information regarding directionality and are more likely to be distorted by crossing fibers, a complementary tractography based connectome analysis was included. The following preprocessing steps have already been described in detail in a previous publication^23^. The publicly available CATO toolbox^24^ was used for anatomical connectome reconstruction. DW images were realigned and corrected for eddy currents and susceptibility artifacts using FSL’s “eddy”^18^. Diffusion tensor estimation was done with the informed RESTORE algorithm^25,26^ that removes outliers during the fitting, hence reducing the impact of physiological noise artifacts. White matter pathways were reconstructed using deterministic streamline tractography based on the Fiber Assignment by Continuous Tracking (FACT) algorithm^27^. Eight seeds were started per voxel. A tractography streamline was constructed for each seed by following the main diffusion direction from voxel to voxel. Stop criteria included making a sharp turn of >45°, reaching grey matter or exiting the brain mask, of if a voxel had an FA value <.10. We employed a basic single-tensor DTI reconstruction combined with simple deterministic fiber tracking instead of more advanced reconstruction methods (e.g. probabilistic fiber tracking), to provide a sufficient balance between false negative and false positive fiber reconstructions, which is known to have a major impact on network analyses^28^. For each participant the anatomical brain network consisting of 114 areas that reflect a subdivision of the cortical areas from the FreeSurfer’s Desikan-Killiany atlas^29,30^ was reconstructed. Subcortical brain regions were omitted due to poorer DWI signal-to-noise ratio and the dominant effect of subcortical regions on network properties, as we have done in previous work^31,32^. A network connection was defined as at least three tractography streamlines that connect two nodes (i.e., brain regions). This threshold balances sensitivity and specificity of the resulting connectivity matrices^33,34^. Each subject’s network was then stored in a connectivity matrix with rows and columns representing cortical brain regions, and matrix entries representing edges, i.e., connectivity strength measured by the mean FA for all connecting streamlines. For quality control the number of streamlines (NOS) and streamline density (SD; the number of streamlines between two regions divided by their average volume) were computed. Four criteria were used to assess data quality: 1. average number of streamlines, 2. average fractional anisotropy, 3. average prevalence of each participants’ connections (low value, if the participant has “odd” connections), and 4. average prevalence of each participants connected brain regions (high value, if the participant misses commonly found connections). For each of the four criteria, the quartiles (Q1, Q2, Q3) and the interquartile range (IQR=Q3-Q1) were calculated over the sample. Participants were excluded if their value was below Q1-1.5*IQR or above Q3+1.5*IQR. This resulted in the exclusion of N=104 participants.

**Network-Based Statistics (NBS).** Detailed steps of this analysis were described in an earlier study^31^. We used Network-Based Statistics (NBS)^35^ to identify an effect at a cluster-level by performing mass univariate testing at the edge level. First, an ANCOVA was calculated for each edge with the FA values as dependent variable and GCP as independent variable as well as the usual covariates (diagnosis, diagnosis x GCP interaction, age, sex, TIV, Marburg pre body-coil, Marburg post body-coil, IQ_MVT_, and education years). Second, significant edges were identified using a restrictive threshold of F=3.00^35^ to focus on the most strongly associated connections. Permutation testing using 5000 permutations was performed on the supra-threshold connections to ascribe a *p*-value to each cluster (i.e. number of interconnected nodes/brain regions) based on its size. The threshold of significance was set to be *p_FWE_*<.05 to adjust for alpha-inflation.

**T_1_ data acquisition.** At both scanning sites a 3D T_1_ weighted magnetization-prepared rapid acquisition gradient echo (MPRAGE) sequence was measured with slightly different scanner protocols (RT 1900ms; Marburg: ET 2.26ms / Münster ET 2.28ms, inversion time 900ms, Marburg: flip-angle 9° / Münster: flip-angle 8°, Marburg: 176 sagittal slices with 0.5mm slice gap / Münster: 192 sagittal slices with 0mm slice gap, 1x1x1mm³ voxel size). A previous study discussed possible site differences due to different scanners and acquisition protocols of the FOR2107-cohort and suggested to correct for those differences using dummy-coded covariates^36^.

**T_1_ image preprocessing.** To corroborate previous results, we repeated the analysis described above in T_1_ structural data. Positive associations between grey matter volume and cognitive performance in the prefrontal cortex, the hippocampus, the anterior cingulate cortex, and the basal ganglia were expected.

We conducted a voxel-based morphometry (VBM) analysis. Structural data was processed with the Computational Anatomy (CAT12) toolbox (version 12.7 r1720, http://dbm.neuro.uni-jena.de/cat/) for the Statistical Parametric Mapping (SPM12) software with default parameters. Preprocessing steps included segmentation into grey matter, white matter, and cerebrospinal fluid and spatial normalization into MNI space using the Geodesic Shooting algorithm. Finally, the grey matter segments were smoothed with a Gaussian filter (8mm full-width half-maximum, FWHM). For quality control, the homogeneity function of CAT12 was used and outliers were carefully visually inspected. One HC and one MDD participant had to be excluded due to inadequate image quality.

**T_1_ voxel-based morphometry (VBM) analysis.** An ANCOVA with grey matter values as independent variable, diagnosis (HC vs. MDD), the extracted neurocognitive factors, and their interaction with diagnosis as independent variables, and age, sex, TIV, Marburg pre body-coil, Marburg post body-coil, IQ_MVT_ and number of education years as nuisance variables was calculated. A whole-brain analysis was conducted with a significance level of *p_tfce-FWE_*<.05.

**Supplement 4: Statistics and general methods**

Post-hoc power-analyses were conducted in G*Power 3.1.9.7 with an exploratory threshold of α<.001. For the group differences (MDD vs. HC) a small effect size of *d*=.26^37^ was assumed resulting in a moderate statistical power of 0.79. For the main effect of GCP a small to medium effect size of *d*=.43^38^ was expected, resulting in a high statistical power of 0.99. The entire sample of *N*=1 007, hence, was an adequate tradeoff to detect both effects of interest. The assumptions of an ANCOVA were tested prior to the data analysis: Measurements were independent, error variances were similar between diagnosis groups (*Levene-F*(11,995)=.74, *p*=.697), homoscedasticity was present (*χ²*(57)=64.49, *p*=.231) and no relevant outliers were present (*Cooks’ distance:* *M*=0.001, *SD*=0.002, *range* ]0.000, 0.018]). All tests are one-sided.

**Supplement 5: Effects in MD, AD and RD**

**Replication of group differences (MDD vs. HC).** Prior to the inclusion GCP in the model, we found a significant main effect of diagnosis in AD (*p_FWE_*=.016, k= 4015 voxels in 5 cluster, x = 20, y = 45, z=18, Supplementary Table 1). MDD patients had lower AD values compared with HC in the forceps minor, the anterior thalamic radiation and the cingulum (Supplementary Table 2). There were no significant effects for MD and RD (all *p_FWE_*>.191).

**ANCOVA including the general cognitive performance factor (GCP).** After including GCP into the model, we found a significant main effect of diagnosis for AD (*p_FWE_*=.010, k=12 009 voxels in 4 cluster, MNI-coordinates of the most significant peak voxel: x=21, y=45, z=18, Supplementary Table 1) in the forceps minor and superior longitudinal fasciculus among other regions (Supplementary Table 2), while no effect was found for MD (*p_FWE_*=.074) and RD (*p_FWE_*=.221). Further, a significant main effect of GCP was found for MD (*p_FWE_*=.006, k=41 080 voxels in 2 clusters, MNI-coordinates of the most significant peak voxel: x=-19, y=-26, z=37) and RD (*p_FWE_*=.001, k=45 963 voxels in 2 clusters‬, MNI-coordinates of the most significant voxel: x=-20, y=-54, z=22) in the forceps minor and the superior longitudinal fasciculus among other regions (Supplementary Table 1 and 2), while no effect was found for AD (*p_FWE_*=.183). No diagnosis x GCP interaction was found (all *p_FWE_*>.215).

**Supplement 6: Principal component analysis (PCA)**

The following twelve tests were included in the PCA: TMT-A, TMT-B, DSST, d2, RAVLT-S, RAVLT-R, CBTT-f, CBTT-b, LNS, VF-C, VF-P and VF-A. The tests could be allocated to five predefined components after a varimax rotation: 1. executive functioning and sustained attention (TMT-A: -.802, TMT-B: -.756, DSST: .719, d2: .676) with an eigenvalue of 4.953, explaining 41.27% of the variance (α=-.678); 2. semantic processing (VF-C: .769, VF-P: .749, VF-A: .730) with an eigenvalue of 1.289, explaining 10.72% of the variance (α=.658); 3. memory (RAVLT-S: .896, RAVLT-R: .765) with an eigenvalue of 1.009, explaining 8.41% of the variance (α=.461); 4. visuospatial working memory (CBTT-f: .853, CBTT-b: .745) with an eigenvalue of 0.810, explaining 6.75% of the variance (α=.679); and lastly, 5. verbal working memory (LNS: .872) with an eigenvalue of 0.720, explaining 6.00% of the variance.

**Supplement 7: Excluding (partially) remitted MDD patients from the analysis**

In a supplementary analysis adressing the role of remission status, (partially) remitted MDD patients were excluded from the analysis. Likewise, we found a positive association of FA with GCP in acute MDD and HC (*p_tfce-FWE_*=.003, k=40 957 voxels in 3 clusters, MNI-coordinates of the peak voxel: x=17, y=37, z=11). Again, no main effect of diagnosis (*p_tfce-FWE_*=.100) nor a diagnosis x GCP interaction (*p_tfce-FWE_*=.344) could be found.

**Supplement 8: NBS analysis**

NBS analysis showed a positive association of FA and GCP in a subnetwork consisting of 283 edges and 112 nodes (see Supplementary Figure 1) at a significance level of *p_FWE_*<.05 (F-threshold 3.00). This subnetwork comprised connections from nearly all brain regions (the anatomical atlas used included 114 nodes total), confirming that the effect was wide-spread and anatomically non-specific. The ANCOVA revealed a significant main effect of GCP (*p*=.029). The main effect of diagnosis (*p*=.689) or the diagnosis x GCP interaction (*p*=.147) were not significant.

**Supplement 9: Supplementary Tables**

| *Supplementary Table 1.*  Factor-loadings of the neurocognitive test battery on the general cognitive performance factor (GCP) | |
| --- | --- |
| Neurocognitive test | Factor loadings |
| The Digit Symbol Substitution Test, DSST | .777 |
| The d2 Test of Attention, d2 | .767 |
| The Trail-Making-Test-B, TMT-B | -.745 |
| The Rey Auditory Verbal Learning Test, overall, RAVLT-S | .701 |
| The Trail-Making-Test-A, TMT-A | -.693 |
| The Corsi block-tapping test, backwards, CBTT-b | .658 |
| The German version of the Letter-Number-Sequences, LNS | .606 |
| The Corsi block-tapping test, forwards, CBTT-f | .578 |
| The Verbal Fluency Test, category, VF-C | .548 |
| The Verbal Fluency Test, alternating, VF-A | .548 |
| The Rey Auditory Verbal Learning Test, recognition, RAVLT-R | .537 |
| The Verbal Fluency Test, category, phonemic, VF-P | .461 |

| *Supplementary Table 2.*  Anatomical locations of the “JHU White-Matter Tractography Atlas” of the significant effects estimated with “atlasquery”. The numbers represent the average probability of the mask of the significant cluster to be a member of the different labelled regions within the atlas. | | | | | |
| --- | --- | --- | --- | --- | --- |
| Region | Side | FA | AD | MD | RD |
| Main effect of diagnosis, prior to the inclusion of the general cognitive performance factor | | | | | |
| Anterior thalamic radiation | L/R | 0.16/1.12* | 1.11/2.48 | - | - |
| Corticospinal tract | L/R | 1.22/1.25* | -/0.99 | - | - |
| Cingulum (cingulate gyrus) | L/R | 0.29/0.07* | 1.27/0.06 | - | - |
| Cingulum (hippocampus) | L/R | >0.01/0.03* | - |  |  |
| Forceps major | B | 1.87* | 0.54 | - | - |
| Forceps minor | B | 4.37* | 24.92 | - | - |
| Inferior fronto-occipital fasciculus | L/R | 1.70/2.35* | 0.92/1.05 | - | - |
| Inferior longitudinal fasciculus | L | 1.77/1.77* | 0.14 | - | - |
| Superior longitudinal fasciculus | L/R | 2.68/3.20* | 0.02/0.38 | - | - |
| Superior longitudinal fasciculus (temporal part) | L/R | 1.16/1.10* | 0.05/0.06 | - | - |
| Uncinate fasciculus | L/R | 0.01/0.02* | 0.63/0.71 | - | - |
| Main effect of diagnosis, after the inclusion of the general cognitive performance factor | | | | | |
| Anterior thalamic radiation | L/R | - | 0.47/1.41 | - | - |
| Corticospinal tract | R | - | 1.15 | - | - |
| Cingulum (cingulate gyrus) | L/R | - | 0.51/0.13 | - | - |
| Cingulum (hippocampus) | L/R | - | <0.01/<0.01 | - | - |
| Forceps major | B | - | 0.28 | - | - |
| Forceps minor | B | - | 10.28 | - | - |
| Inferior fronto-occipital fasciculus | L/R | - | 0.43/2.15 | - | - |
| Inferior longitudinal fasciculus | L/R | - | 0.07/0.38 | - | - |
| Superior longitudinal fasciculus | L/R | - | <0.01/4.18 | - | - |
| Uncinate fasciculus | L/R | - | 0.29/0.66 | - | - |
| Superior longitudinal fasciculus (temporal part) | L/R | - | 0.01/1.29 | - | - |
| Main effect of the general cognitive performance factor | | | | | |
| Anterior thalamic radiation | L/R | 1.77/1.37 | - | 1.0/0.93 | 1.15/1.21 |
| Corticospinal tract | L/R | 0.87/0.85 | - | 1.12/0.83 | 1.03/0.78 |
| Cingulum (cingulate gyrus) | L/R | 0.37/0.11 | - | 0.34/0.06 | 0.40/0.10 |
| Cingulum (hippocampus) | L/R | 0.02/0.10 | - | 0.01/0.02 | 0.02/0.10 |
| Forceps major | B | 1.21 | - | 0.52 | 0.84 |
| Forceps minor | B | 2.53 | - | 2.40 | 2.73 |
| Inferior fronto-occipital fasciculus | L/R | 1.99/1.88; (-/3.67)** | - | 1.69/1.77 | 1.88/1.87 |
| Inferior longitudinal fasciculus | L/R | 1.88/1.21; (-/12.33)** | - | 1.61/1.12 | 1.80/1.27 |
| Superior longitudinal fasciculus | L/R | 2.15/1.69; (-/2.67)** | - | 2.42/2.40 | 2.43/2.08 |
| Uncinate fasciculus | L/R | 0.55/0.19 | - | 0.68/0.30 | 0.59/0.25 |
| Superior longitudinal fasciculus (temporal part) | L/R | 0.97/0.56; (0/3.00)* | - | 1.13/0.90 | 1.10/0.76 |
| *Note:* *this effect was not significant in the entire sample but only after the exclusion of partially remitted MDD patients; **results for voxel-wise FWE-correction. *Abbreviation:* AD=axial diffusivity, B=bilateral DSST= Digit Symbol Substitution Test, FA=fractional anisotropy, L=left, MDD=Major Depressive Disorder, MD=mean diffusivity, R=right, RD=radial diffusivity. | | | | | |

| *Supplementary Table 3.*  Location and size of all significant clusters. | | | | | |
| --- | --- | --- | --- | --- | --- |
| Cluster / contrast | *p_FWE_* | k | x | y | z |
| Fractional anisotropy |  |  |  |  |  |
| Main effect of the processing speed factor; pos. correlation |  |  |  |  |  |
| 1. Cluster | .002 | 43 700 | -31 | -66 | 11 |
| Axial diffusivity |  |  |  |  |  |
| HC > MDD, without processing speed factor |  |  |  |  |  |
| 1. Cluster | .016 | 3 516 | 20 | 45 | 18 |
| 1. Cluster | .042 | 298 | 19 | -21 | 52 |
| 1. Cluster | .036 | 100 | -22 | -78 | 14 |
| 1. Cluster | .044 | 84 | 31 | -45 | 35 |
| 1. Cluster | .048 | 17 | 34 | -18 | 38 |
| HC > MDD, with processing speed factor |  |  |  |  |  |
| 1. Cluster | .010 | 11 513 | 21 | 45 | 18 |
| 1. Cluster | .046 | 170 | 30 | -1 | 18 |
| 1. Cluster | .040 | 168 | 41 | -36 | -10 |
| 1. Cluster | .035 | 158 | -22 | -78 | 14 |
| Mean diffusivity |  |  |  |  |  |
| Main effect of the processing speed factor |  |  |  |  |  |
| 1. Cluster | .006 | 41 058 | -19 | -26 | 37 |
| 1. Cluster | .049 | 22 | -30 | -5 | -31 |
| Radial diffusivity |  |  |  |  |  |
| Main effect of the processing speed factor |  |  |  |  |  |
| 1. Cluster | .001 | 45 907 | -20 | -54 | 22 |
| 1. Cluster | .048 | 56 | -4 | -3 | 3 |
| *Abbreviations:* HC=healthy control, k=voxel count, MDD=Major Depressive Disorder, x-y-z=location in three dimensional Montreal Neurological Institute (MNI) space. | | | | | |

| *Supplementary Table 4.*  An ANCOVA was calculated with the mean extracted FA values as independent variable and the five subdomains of general cognitive performance as described in Supplements 3 as independent variables on top of age, sex, TIV, scanner/side variables, IQ_MVT_ and number of education years in SPSS. | | | |
| --- | --- | --- | --- |
| Factor / Covariate | *F*-statistic, df(1,993) | *p*-value | *η²* |
| Diagnosis | 0.304 | .581 | <.001 |
| Age | 56.569 | <.001 | .054 |
| Sex | 0.079 | .778 | <.001 |
| TIV | 55.616 | <.001 | .053 |
| Marburg pre body-coil | 200.305 | <.001 | .168 |
| Marburg post body-coil | 87.734 | <.001 | .081 |
| IQ_MVT_ | 4.391 | .036 | .004 |
| Number of education years | 2.380 | .123 | .002 |
| Processing speed and sustained attention | 30.116 | <.001 | .029 |
| Memory | 10.794 | .001 | .011 |
| Visuospatial working memory | 9.306 | .002 | .009 |
| Verbal working memory | 2.326 | .128 | .002 |
| Semantic processing | 11.084 | .001 | .011 |
| *Abbreviations:* ANCOVA=Analysis of covariance, FA=fractional anisotropy, IQ_MVT=_ Intelligence quotient evaluated with the multiple-choice vocabulary test version B (dt. “Mehrfachwahl-Wortschatz-Test Version B”), TIV=total intracranial volume | | | |

**Supplement 10: Supplementary Figures**

**Supplementary Figure 1.** Scree-plot of the principal component analysis using all twelve neurocognitive tests. While three components were above the Kaiser criteria of >1, the elbow-criteria clearly hints at a one factor solution.

| 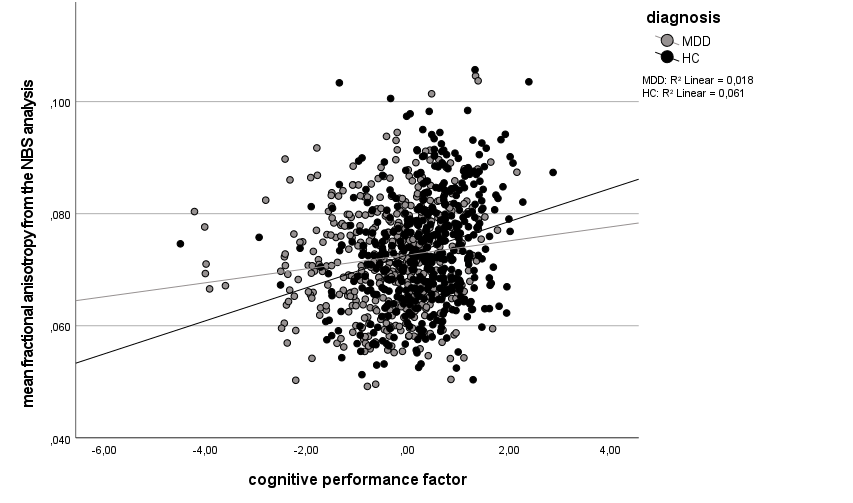 | | |
| --- | --- | --- |
| 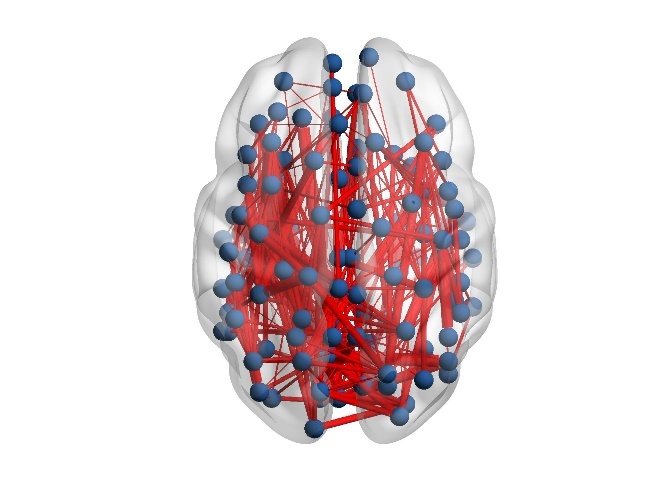 | 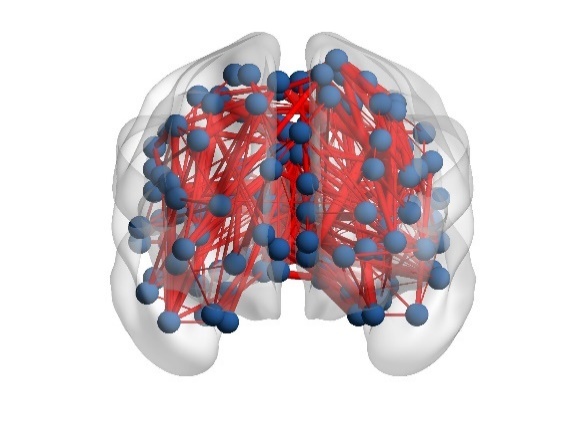 | 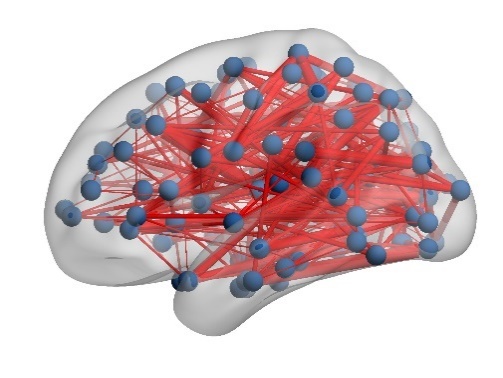 |
| **Supplementary Figure 2.** Edges (red) and nodes (blue) that show a positive association of general cognitive performance and fractional ansisotropy (FA) within the whole sample. Results show a subnetwork based on a network-based-statistics (NBS) analysis with a threshold of *p­_FWE_*<.05 and a suprathreshold F-value of F=3.00. Images were created using the BrainNet Viewer software^39^. Top: scatterplot depicting the association of mean FA (extracted from the significant cluster), left: axial view, middle: coronal view, right: sagittal view. | | |

Supplementary References

1 Bettcher BM, Libon DJ, Kaplan E, Swenson R, Penney DL. Digit Symbol Substitution Test. In Kreutzer JS, Caplan B, DeLuca J (eds). *Encyclopedia of clinical neuropsychology*. Springer reference. Springer: New York, London, 2011, pp. 849–853.

2 Wechsler D. *Wechsler adult intelligence scale. Administration and Scoring Manual TPC*. New York: Oxford University Press, 1997.

3 Tewes U. Hamburg-Wechsler-Intelligenztest für Erwachsene-rev. ed. *HAWIE-R). Bern, Stuttgart, Toronto: Huber* 1991.

4 Corrigan JD, Hinkeldey NS. Relationships between parts A and B of the Trail Making Test. *Journal of clinical psychology* 1987; **43**: 402–409.

5 Gaudino EA, Geisler MW, Squires NK. Construct validity in the Trail Making Test: what makes Part B harder? *Journal of clinical and experimental neuropsychology* 1995; **17**: 529–535.

6 Ross RM. *The d2 test of attention: An examination of age, gender, and cross-cultural indices*. Argosy University, 2005.

7 Britt DM, Adams Jr SG, Godding PR, Grothues CA, Varnado P. Clinical differentiation of the Rey Auditory-Verbal Learning Test. *American Journal of Alzheimer’s Disease* 1995; **10**: 7–18.

8 Rosenberg SJ, Ryan JJ, Prifitera A. Rey auditory-verbal learning test performance of patients with and without memory impairment. *Journal of clinical psychology* 1984; **40**: 785–787.

9 Kessels RPC, van den Berg E, Ruis C, Brands AMA. The backward span of the Corsi Block-Tapping Task and its association with the WAIS-III Digit Span. *Assessment* 2008; **15**: 426–434.

10 Kessels RP, van Zandvoort MJ, Postma A, Kappelle LJ, Haan EH de. The Corsi Block-Tapping Task: standardization and normative data. *Applied neuropsychology* 2000; **7**: 252–258.

11 Shao Z, Janse E, Visser K, Meyer AS. What do verbal fluency tasks measure? Predictors of verbal fluency performance in older adults. *Frontiers in psychology* 2014; **5**.

12 Lehrl S, Triebig G, Fischer B. Multiple choice vocabulary test MWT as a valid and short test to estimate premorbid intelligence. *Acta neurologica Scandinavica* 1995; **91**: 335–345.

13 Lehrl S. *Mehrfachwahl-Wortschatz-Intelligenztest: MWT-B,* 5th edn. Spitta: Balingen, 2005.

14 Oguz I, Farzinfar M, Matsui J, Budin F, Liu Z, Gerig G *et al.* DTIPrep: quality control of diffusion-weighted images. *Frontiers in neuroinformatics* 2014; **8**: 4.

15 Jenkinson M, Beckmann CF, Behrens TEJ, Woolrich MW, Smith SM. FSL. *NeuroImage* 2012; **62**: 782–790.

16 Smith SM, Jenkinson M, Woolrich MW, Beckmann CF, Behrens TEJ, Johansen-Berg H *et al.* Advances in functional and structural MR image analysis and implementation as FSL. *NeuroImage* 2004; **23 Suppl 1**: S208-19.

17 Woolrich MW, Jbabdi S, Patenaude B, Chappell M, Makni S, Behrens TEJ *et al.* Bayesian analysis of neuroimaging data in FSL. *NeuroImage* 2009; **45**: S173-86.

18 Andersson JLR, Sotiropoulos SN. An integrated approach to correction for off-resonance effects and subject movement in diffusion MR imaging. *NeuroImage* 2016; **125**: 1063–1078.

19 Smith SM. Fast robust automated brain extraction. *Human brain mapping* 2002; **17**: 143–155.

20 Hua K, Zhang J, Wakana S, Jiang H, Li X, Reich DS *et al.* Tract probability maps in stereotaxic spaces: analyses of white matter anatomy and tract-specific quantification. *NeuroImage* 2008; **39**: 336–347.

21 Mori S, Wakana S, van Zijl PCM, Nagae-Poetscher LM. *MRI Atlas of Human White Matter,* 1st edn. Elsevier: Amsterdam, The Netherlands, 2005.

22 Wakana S, Caprihan A, Panzenboeck MM, Fallon JH, Perry M, Gollub RL *et al.* Reproducibility of quantitative tractography methods applied to cerebral white matter. *NeuroImage* 2007; **36**: 630–644.

23 Collin G, van den Heuvel MP, Abramovic L, Vreeker A, Reus MA de, van Haren NEM *et al.* Brain network analysis reveals affected connectome structure in bipolar I disorder. *Human brain mapping* 2016; **37**: 122–134.

24 Lange SC de, van den Heuvel MP. *Structural and functional connectivity reconstruction with CATO - A Connectivity Analysis TOolbox*, 2021.

25 Chang L-C, Jones DK, Pierpaoli C. RESTORE: Robust estimation of tensors by outlier rejection. *Magnetic resonance in medicine* 2005; **53**: 1088–1095.

26 Chang L-C, Walker L, Pierpaoli C. Informed RESTORE: A method for robust estimation of diffusion tensor from low redundancy datasets in the presence of physiological noise artifacts. *Magn Reson Med* 2012; **68**: 1654–1663.

27 Mori S, van Zijl PCM. Fiber tracking: principles and strategies - a technical review. *NMR in biomedicine* 2002; **15**: 468–480.

28 Sarwar T, Ramamohanarao K, Zalesky A. Mapping connectomes with diffusion MRI: deterministic or probabilistic tractography? *Magnetic resonance in medicine* 2019; **81**: 1368–1384.

29 Hagmann P, Cammoun L, Gigandet X, Meuli R, Honey CJ, van Wedeen J *et al.* Mapping the structural core of human cerebral cortex. *PLoS biology* 2008; **6**: e159.

30 Cammoun L, Gigandet X, Meskaldji D, Thiran JP, Sporns O, Do KQ *et al.* Mapping the human connectome at multiple scales with diffusion spectrum MRI. *Journal of neuroscience methods* 2012; **203**: 386–397.

31 Repple J, Mauritz M, Meinert S, Lange SC de, Grotegerd D, Opel N *et al.* Severity of current depression and remission status are associated with structural connectome alterations in major depressive disorder. *Molecular psychiatry* 2019.

32 Lange SC de, Scholtens LH, van den Berg LH, Boks MP, Bozzali M, Cahn W *et al.* Shared vulnerability for connectome alterations across psychiatric and neurological brain disorders. *Nature human behaviour* 2019; **3**: 988–998.

33 Zalesky A, Fornito A, Cocchi L, Gollo LL, van den Heuvel MP, Breakspear M. Connectome sensitivity or specificity: which is more important? *NeuroImage* 2016; **142**: 407–420.

34 Reus MA de, van den Heuvel MP. Estimating false positives and negatives in brain networks. *NeuroImage* 2013; **70**: 402–409.

35 Zalesky A, Fornito A, Bullmore ET. Network-based statistic: identifying differences in brain networks. *NeuroImage* 2010; **53**: 1197–1207.

36 Vogelbacher C, Möbius TWD, Sommer J, Schuster V, Dannlowski U, Kircher T *et al.* The Marburg-Münster Affective Disorders Cohort Study (MACS): A quality assurance protocol for MR neuroimaging data. *NeuroImage* 2018; **172**: 450–460.

37 van Velzen LS, Kelly S, Isaev D, Aleman A, Aftanas LI, Bauer J *et al.* White matter disturbances in major depressive disorder: a coordinated analysis across 20 international cohorts in the ENIGMA MDD working group. *Molecular psychiatry* 2019.

38 Opel N, Martin S, Meinert S, Redlich R, Enneking V, Richter M *et al.* White matter microstructure mediates the association between physical fitness and cognition in healthy, young adults. *Scientific reports* 2019; **9**: 12885.

39 Xia M, Wang J, He Y. BrainNet Viewer: a network visualization tool for human brain connectomics. *PloS one* 2013; **8**: e68910.
